# Age‐related telomere attrition causes aberrant gene expression in sub‐telomeric regions

**DOI:** 10.1111/acel.13357

**Published:** 2021-05-21

**Authors:** Xiao Dong, Shixiang Sun, Lei Zhang, Seungsoo Kim, Zhidong Tu, Cristina Montagna, Alexander Y. Maslov, Yousin Suh, Tao Wang, Judith Campisi, Jan Vijg

**Affiliations:** ^1^ Department of Genetics Albert Einstein College of Medicine Bronx NY USA; ^2^ Institute on the Biology of Aging and Metabolism Department of Genetics, Cell Biology and Development University of Minnesota Minneapolis MN USA; ^3^ Department of Obstetrics and Gynecology Columbia University Irving Medical Center New York NY USA; ^4^ Department of Genetics and Genomic Sciences Icahn Institute for Genomics and Multiscale Biology Icahn School of Medicine Mount Sinai New York NY USA; ^5^ Laboratory of Applied Genomic Technologies Voronezh State University of Engineering Technology Voronezh Russia; ^6^ Department of Genetics and Development Columbia University Irving Medical Center New York NY USA; ^7^ Department of Epidemiology & Population Health Albert Einstein College of Medicine Bronx NY USA; ^8^ Buck Institute for Research on Aging Novato CA USA; ^9^ School of Public Health Center for Single‐Cell Omics Shanghai Jiao Tong University School of Medicine Shanghai China

**Keywords:** aging, gene expression, telomere shortening

## Abstract

Telomere attrition has been proposed as a biomarker and causal factor in aging. In addition to causing cellular senescence and apoptosis, telomere shortening has been found to affect gene expression in subtelomeric regions. Here, we analyzed the distribution of age‐related differentially expressed genes from the GTEx RNA sequencing database of 54 tissue types from 979 human subjects and found significantly more upregulated than downregulated genes in subtelomeric regions as compared to the genome‐wide average. Our data demonstrate spatial relationships between telomeres and gene expression in aging.

## Graphical abstract

Telomere shortening has been found affecting expression of individual genes in subtelomeric regions through a loss of chromosome looping. Using the GTEx RNA database and published Hi‐C data, we demonstrate that this effect indeed occurs during human aging in many tissues, including those for which age‐related telomere attrition has been demonstrated.

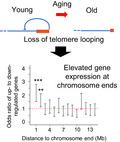

## 1 INTRODUCTION

Telomeres are tracts of repetitive nucleotide sequences that protect chromosome ends from uncapping and causing widespread genome instability (Blackburn et al., 2015; de Lange, 2018). In the absence of the enzyme telomerase from most human somatic cells, telomere repeats shorten as a consequence of the end replication problem (Olovnikov, 1973), oxidative stress (von Zglinicki et al., 1995), and possibly other factors. This telomere shortening is hypothesized to causally contribute to the functional decline and increased disease risk that occurs during aging through cellular senescence and/or cell death (Campisi, 2013; Shay & Wright, 2019). However, it is unclear whether, during normal aging, telomeres become short enough to cause cell death or senescence on a scale sufficient to cause the myriad diseases of aging, much less the late‐life aplastic anemia, pulmonary fibrosis, and hepatic cirrhosis that occurs in telomeropathies (Holohan et al., 2014). However, telomere shortening could contribute to aging without uncapping. Indeed, using isogenic pairs of human myoblasts and fibroblasts in culture, it has been demonstrated that when telomeres are long they interact through chromosome looping with genes up to 10 Mb away from the telomere (Baur et al., 2001; Robin et al., 2014). Such interaction is prevented when telomeres are short but still far from short enough to cause uncapping. Interestingly, this telomere position effect was shown to cause deregulation of genes in the subtelomeric regions, most notably illegitimate overexpression as a consequence of a loss of the contact‐mediated gene repression. This phenomenon has been confirmed for at least one gene, interferon‐stimulated gene 15 (*ISG15*, 1p36.33) which was found upregulated in a fraction of cells in human dermis from elderly subjects (Lou et al., 2009). Here we show, using the GTEx RNA sequencing database, that during aging in a variety of tissues telomere shortening does indeed lead to deregulation of gene expression in subtelomeric regions.

## 2 RESULTS

**FIGURE 1:**
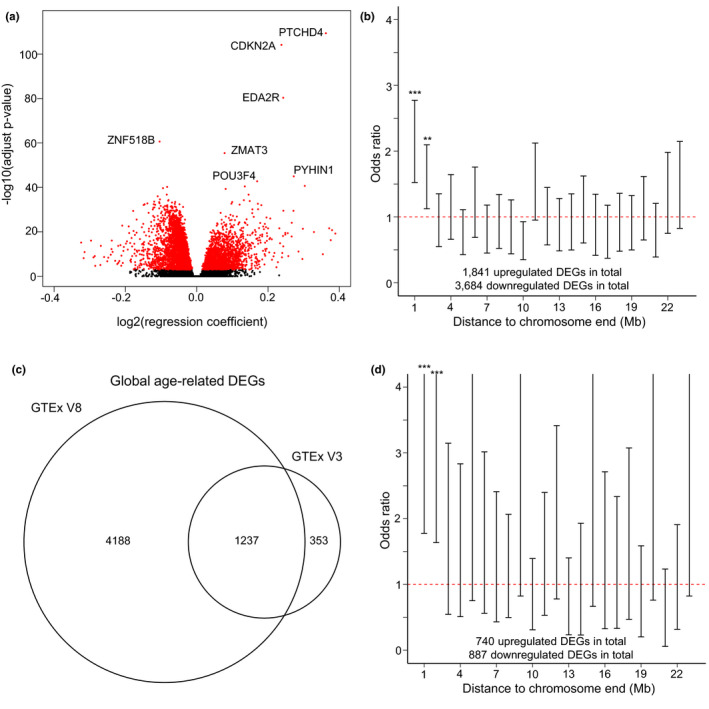
Age‐related DEGs as a function of their distance to chromosome ends. (a) Volcano plot displaying global age‐related DEGs discovered across all tissue types using the GTEx (version 8). (b) Age‐related DEGs as a function of their distance from the chromosome ends (GTEx version 8). (c) Venn plot showing the overlap between results in the GTEx version 8 and the GTEx version 3 in the global analysis. 78% of DEGs reported in the GTEx version 3 overlapped with the GTEx version 8. (d) Age‐related DEGs as a function of their distance from the chromosome ends (GTEx version 3). Odds ratio was calculated as (No. upregulated DEG in the bin ×No. downregulated DEG elsewhere) / (No. upregulated DEG elsewhere ×No. downregulated DEG in the bin). ***p < 0.001. **p < 0.01. Error bars represent 95% confidence intervals

To analyze the distribution of age‐related differentially expressed genes across chromosomes, we used the GTEx database (version 8), which includes RNA sequencing data of 17,382 samples of 54 tissue types collected from 979 human subjects varying in age from 20 to 79 year (Materials and Methods). We first identified 5,525 global age‐related differentially expressed genes (DEGs) and age‐related DEGs for each of the individual 54 tissue types (Materials and Methods; Figure 1a and Table S1). With gene ontology (GO) enrichment analysis (Materials and Methods), we found these DEGs enriched in 1,176 GO terms (*q* value <0.05), many of them previously documented age‐related pathways, for example, autophagy, mitochondrial function, replicative senescence, TOR signaling, and Wnt signaling (Table S2) (Campisi, 2013; Cuervo, 2008; Kapahi et al., 2010; Liu et al., 2007; Short et al., 2005).

We then spaced the genome into bins of 1 Mb and found that the first and second bins closest to chromosome ends showed significantly higher ratios of upregulated to downregulated DEGs than elsewhere in the genome (*p* = 1.6 × 10^−7^ and 6.2 × 10^−3^ for the first two bins separately, Fisher's exact test, two‐tailed; Figure 1b). To affirm that this effect is telomere‐specific, we performed the same analysis for bins close to centromeres and did not observe the same increase in ratio (*p* = 0.49 and 0.59 for the first two bins separately; Figure S1).

To further confirm our results, we compared the age‐related DEGs found by us with those reported by others using an earlier GTEx version (version 3) (Mele et al., 2015). The results indicate substantial overlap (Figure 1c). The higher number of DEGs in our present study is likely due to the smaller sample size in the previous GTEx version compared to the current version 8. Nevertheless, the same higher ratio of up‐ to downregulated genes in the chromosome end bins was found in the age‐related DEGs reported by Mele et al., (2015) (*p* = 4.5 × 10^−5^ and 3.1 × 10^−4^ for the first two bins separately; Figure 1d).

Next, we tested if the upregulated global age‐related DEGs in the first 2 Mb of chromosome ends were enriched for specific genetic pathways. Using the 174 upregulated genes in these regions (Table S3), we found no enrichment for any of the Gene Ontology terms using the R package “clusterProfiler” (Yu et al., 2012). However, we did observe individual DEGs related to aging and telomere maintenance, including *RAD52* (Verma et al., 2019) (maintaining telomere length during alternative lengthening of telomeres) and *HSF1* (Koskas et al., 2017) (involved in telomere protection upon stress) (Table S3).

**FIGURE 2:**
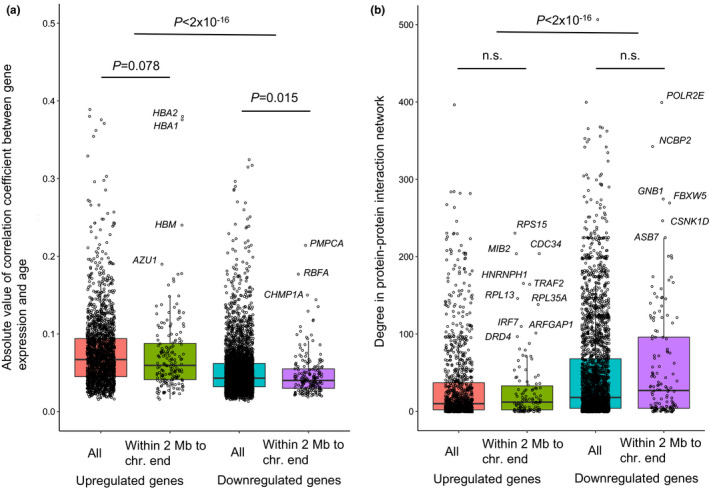
Comparison between upregulated and downregulated DEGs, and between genome‐wide DEGs and DEGs close to chromosome ends. (a) Correlation coefficient between gene expression and age. (b) Network node‐degree of DEG proteins in the PPI network of 5,525 age‐related DEGs. *p* values were estimated using the *Wilcoxon Rank Sum* test, two‐tailed. The “n.s.” indicates “not significant”

We then prioritized all differentially expressed genes according to the correlation coefficient between the change in their expression level and age (Figure 2a). We found this correlation coefficient to be significantly higher for upregulated than for downregulated genes (*p* < 2 × 10^−16^), with a weaker correlation coefficient for genes close to chromosome ends (≤2 Mb; *p* = 0.078 and 0.015 for upregulated and downregulated genes, separately) than those across the genome overall. For genes close to chromosome ends, two genes stood out as being highly upregulated, that is, hemoglobin A2 (*HBA2*) and hemoglobin A1 (*HBA1*). Together with the third highest, Mu hemoglobin (*HBM*) gene, these are all hemoglobin subunit genes, expressed almost exclusively in blood and bone marrow, which may simply reflect a higher sensitivity in identifying age‐related DEGs from blood, the tissue type with the second largest sample size in the GTEx data set, as compared to other tissues.

Next, we analyzed all DEGs for interactions in a protein–protein interaction (PPI) network, that is, the STRING database (quality score ≥0.9) (Szklarczyk et al., 2019). A subnetwork was extracted from the global PPI network by selecting only the proteins corresponding to the 5,525 DEGs. We then prioritized the 5,525 DEG proteins according to their degree in the subnetwork. The degree value of a protein is the number of proteins the index protein interacts with in the subnetwork. We compared degrees between upregulated and downregulated genes as well as between all DEG proteins and those close to chromosome ends (≤2 Mb). As shown in Figure 2b, we found that across the entire genome, the upregulated gene proteins show weaker degrees of interaction in the subnetwork than the downregulated gene proteins. However, when only looking at DEG proteins from genes close to chromosome ends (≤2 Mb), no such difference was found. We examined some DEG proteins, corresponding to genes close to telomeres, in further detail based on their high degrees of interaction. Interferon regulatory factor 7 (*IRF7*), a member of the interferon regulatory factor family of transcription factors, is upregulated with increased age, whose overexpression could lead to senescence in immortal fibroblasts (Li et al., 2008). Another gene of potential interest is Dopamine Receptor D4 (*DRD4*) with a correlation coefficient of 0.178, which may influence longevity and physical activity in the mouse (Grady et al., 2013).

In spite of the elevated ratio of upregulated versus downregulated DEGs in subtelomeric regions, the results above indicate that DEGs at chromosome ends are not more strongly correlated with age than genes located elsewhere in the genome. Of note, this does not exclude the possibility that some individual DEGs in these regions could have an important effect during aging.

**FIGURE 3:**
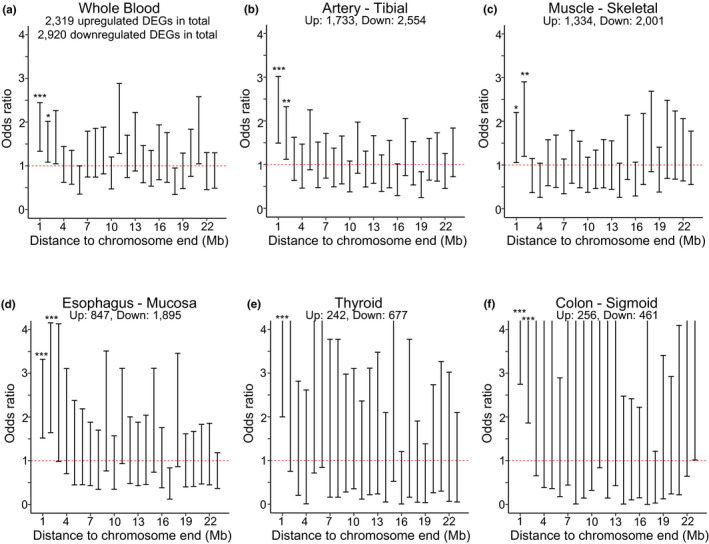
Odds ratio of age‐related upregulated and downregulated DEGs in for the six significant tissue types. Odds ratio was calculated as (No. upregulated DEG in the bin ×No. downregulated DEG elsewhere) / (No. upregulated DEG elsewhere ×No. downregulated DEG in the bin). ***p < 0.001. **p < 0.01. *p < 0.05. Error bars represent 95% confidence intervals. Figures are sorted according to the total numbers of DEGs identified in each tissue. *p* values for the first two bins are 8.0 × 10^−5^ and 1.1 × 10^−2^ in whole blood, 1.3 × 10^−5^ and 8.5 × 10^−3^ in tibial artery, 2.2 × 10^−2^ and 4.5 × 10^−3^ in skeletal muscle, 3.2 × 10^−5^ and 2.9 × 10^−5^ in esophagus mucosa, 1.1 × 10^−4^ and 1.6 × 10^−1^ in thyroid, and 2.3 × 10^−6^ and 2.8 × 10^−4^ in sigmoid colon

We then analyzed age‐related DEGs of each tissue type separately, again using GTEx version 8. Despite a significant overlap, many tissue types had their own specific age‐related DEGs (Figure S2). For several tissue types, we could not identify any age‐related DEGs, likely due to the limited sample size. Using this data, we found that six tissue types showed significantly increased ratios of upregulated to downregulated genes in the first two bins (Figure 3a‐f). In all these six tissue types, that is, whole blood, tibial artery, skeletal muscle, esophagus mucosa, thyroid, and sigmoid colon, telomere shortening with age has been previously observed (Chang & Harley, 1995; Daniali et al., 2013; Demanelis et al., 2020; Takubo et al., 2010). Although no significantly increased ratios in the first two bins were found in the other tissue types, we cannot exclude the possibility that this is due, at least in part, to an insufficient number of DEGs identified for the analysis for each tissue type separately (Figure S3; Table S1).

We then tested for chromosomal looping in subtelomeric regions, which has been found associated with the effect of telomere attrition on gene expression in these regions (Robin et al., 2014), using published Hi‐C (chromosome conformation capture followed by high‐throughput sequencing) data (Dekker et al., 2017). The data set is composed of 22 human cell lines of different cell types and tissues (Table S4). Of note, this data set has no aging component and many cell lines are immortalized cancer cell lines, which may have different chromatin structure and telomere interaction frequency as compared to normal cells. We found that among the chromosome loops with one end locating at the 1 Mb bin closest to the chromosome ends, 86.3% had their other ends anchored within 2 Mb in the subtelomeric regions (Figure S4). As expected, this type of looping was observed across all chromosome ends (Table S5). In addition, but more rarely, we also observed chromosome looping between the first 1 Mb bin and locations up to 11 Mb from the chromosome ends (Figure S4 and Table S6), such as 5q and 19p in HeLa‐S3 and HepS2 cells. This is consistent with previous findings (Kim & Shay, 2018; Robin et al., 2014) and indicates that telomere position effects can occasionally reach much further than the subtelomeric regions. However, a caveat here is that these two cell lines, in which we observed the long interactions, were analyzed using the in situ (in‐nucleus) Hi‐C method, while the other 20 cell lines were analyzed using the dilution (in solution) Hi‐C method. The in situ Hi‐C reduces the frequency of interchromosomal contacts from random ligation events that occur in solution, making it less noisy than dilution Hi‐C (Kempfer & Pombo, 2020). For this reason, we performed the same analysis on an additional Hi‐C data set obtained using the in situ method (Table S4 and S5) (Rao et al., 2014). The results showed 93.8% of chromosome loops linking chromosome ends to the subtelomeric regions, confirming the results obtained from the Dekker et al's Hi‐C data (i.e., the 86.3%, as mentioned).

Overall these results indicate substantial chromosomal looping connections within the first 2 Mb at chromosome ends. Taken together with the results above, this suggests that the effects on the expression of genes in the first 2 Mb from the chromosome ends may be due to loss of looping, exactly as reported for the isogenic cell lines with short and long telomeres (Robin et al., 2014).

## 3 DISCUSSION

Telomere shortening is considered one of the hallmarks of aging and has been implicated as a causal mechanism of age‐related phenotypes through cellular senescence and/or apoptosis (Campisi, 2013; Shay & Wright, 2019; Vijg, 2007). However, apart from telomeropathies there is very little evidence that during normal aging telomeres ever become short enough to uncap on a scale so widespread to cause significant degenerative effects. The previously observed effect of telomere shortening per se, in the absence of uncapping, on gene expression in the subtelomeric regions due to the loss of looping from telomeres (Robin et al., 2014) offers a possible alternative mechanism for aging effects of telomere attrition. Evidence of telomere position effects was also identified in other recent studies, for example, in T cells during healthy aging (Tedone et al., 2019). Here we demonstrated, on a much larger scale using the GTEx database, that the reported telomere position effect indeed occurs during human healthy aging in many of the tissues for which age‐related telomere attrition has been demonstrated (Takubo et al., 2010). We cannot exclude the possibility that a position effect is present in other tissues as well due to an insufficiently high number of DEGs in some of the individual tissue types. Using a Hi‐C database, we also confirmed substantial chromosomal looping connections within the first 2 Mb at chromosome ends, which supports the observed regulatory interactions between telomeres and the deregulated genes in the subtelomeric regions previously reported.

An important question is the relative causal contribution of the telomere position effect to aging. First of all, our data do not rule out that the cellular phenotypes associated with telomere uncapping, that is, apoptosis and cellular senescence, can occur. Indeed, any uncapping would most likely lead to loss of the cell, an event that is very difficult to detect unless it would be so widespread that it cannot be missed, as in telomeropathies or TERT‐deficient mice where an increase in chromosome ends lacking detectable telomere signal by FISH has been observed (Rajaraman et al., 2007). While apoptosis under normal conditions is very rare, with the apoptotic response even further reduced with age (Suh et al., 2002), there is evidence for the accumulation of senescent cells, albeit the frequencies observed are also low and difficult to ascertain due to a lack of reliable markers (Liu et al., 2019). Still, even very small numbers of senescent cells could causally contribute to aging through the senescence‐associated secretory phenotype (Campisi, 2013). In addition, other chromosome end structures, for example, interstitial telomere loops mediated through TRF2 and lamin, could also contribute to aging by altering the expression of subtelomeric genes, even leading to premature aging disorders (Mukherjee et al., 2018; Wood et al., ,^2014^, ^2015^). Of course, a possible causal contribution to the aging process of either telomere uncapping or the telomere position effect on gene expression in subtelomeric regions would be limited to those tissues that show age‐related telomere shortening, which excludes brain and myocardium (Takubo et al., 2010).

In this present study, we did not observe any upregulated global age‐related DEGs in the subtelomeric region to affect specific functional pathways. We also did not find candidate genes that through alteration in their expression could explain aspects of the aging process. However, this does not exclude the possibility that at least some of these genes play a pivotal role in promoting aging and age‐related diseases. Meanwhile, the possibility should be considered that telomere position effects on gene expression in subtelomeric regions are merely random. Such random effects could causally contribute to aging, for example, through increased transcriptional noise (Bahar et al., 2006; Levy et al., 2020).

## 4 EXPERIMENTAL PROCEDURES

### 4.1 Bulk RNA sequences, data resources, and analyses

Raw gene read counts were downloaded from https://www.gtexportal.org/ (version V8, genome build GRCh38, gene annotation GENCODE v26). Genes with average read count <1 were removed. Gene raw counts were normalized by TMM methods in edgeR (Robinson et al., 2010). To identify age‐related differentially expressed genes, we used mixed effect linear regression model for global analysis, and linear regression model for tissue‐specific analyses using DREAM (Hoffman & Roussos, 2020). For the global analysis, we set tissue and sex as fixed factors, age as a covariate and individual as a random effect. For the tissue‐specific analyses, we set age as a covariant, and sex as a factor when samples of both sexes were available. *p* values were corrected using the Bonferroni correction, and age‐related differentially expressed genes (DEGs) were determined if their Bonferroni‐correct *p *< 0.001.

We used protein‐coding genes in 1–22 and X chromosome for further analyses. Distances to the chromosome end were calculated as genes to the last base pair position of closest chromosome end. All genes were grouped using 1 Mb non‐overlapping sliding window (i.e., bin). For each bin, we calculated the odds ratio as (No. upregulated DEG in the bin ×No. downregulated DEG elsewhere) / (No. upregulated DEG elsewhere ×No. downregulated DEG in the bin) and tested its significance using Fisher's exact test. GO enrichment of the DEGs was performed using clusterProfiler (universe: all protein‐coding genes (within 2 Mb if testing genes in first 2 Mb); ont: ALL; pAdjustMethod: BH; qvalueCutoff: 0.1) (Yu et al., 2012).

### 4.2 Hi‐C data analysis

Hi‐C matrices (hic format) were downloaded from the website of the 4DN project (https://data.4 dnucleome.org/) (Dekker et al., 2017). To control the variation of data, we only selected those collected from ENCODE including 22 cell types and tissues, and in situ Hi‐C from Rao et al., (2014) including 4 cell types (Table S4). Data in the Hi‐C matrices include contact matrices under thirteen resolutions, representing connections between any two chromosome loci. The loops were then called using the hiccups function in Juicer (version 1.6) with default setting (Durand et al., 2016). The distances to chromosomal end of loops were integrated and then plotted with Circos (version 0.69.6) (Krzywinski et al., 2009).

## CONFLICT OF INTEREST

XD, LZ, AYM, and JV are co‐founders and stockholders of SingulOmics, Corp. JC is a founder and stockholder in Unity Biotechnology. The other authors declare no competing interests.

## AUTHOR CONTRIBUTIONS

XD and JV conceived and supervised the study. SS analyzed the data, and XD, SS, and JV interpreted the data and wrote the first draft. All authors contributed to editing the manuscript.

## Supporting information

Supplementary Material

Figure S1. Age‐related DEGs as a function of their distance to centromeres.
Figure S2. Overlap of age‐related DEGs between global and tissue‐specific analysis.
Figure S3. Odds ratio of age‐related upregulated and downregulated DEGs in for each tissue types.
Figure S4. Hi‐C loops fromtelomeres mostly end within 2Mb from chromosome ends.

Table S1. Age‐related DEGs calculated from GTEx.
Table S2. Gene‐ontology (GO) enrichment analysis on all DEGs of global analysis.
Table S3. Upregulated global age‐related DEGs in the first 2 Mb of chromosome ends.
Table S4. Basic information of Hi‐C data.
Table S5. Chromosome loops with one end locating at the 1Mb bin closest to chromosome ends.

## Data Availability

For the analyses of age‐related DEGs, raw gene read counts based on RNA sequencing were downloaded from https://www.gtexportal.org/ (version V8, genome build GRCh38, gene annotation GENCODE v26). For the analyses of chromosome conformation at telomere ends, Hi‐C matrices (hic format) were downloaded from the website of the 4DN project (https://data.4dnucleome.org/) (Dekker et al., 2017).
