# Supplementary material for: Age‐related telomere attrition causes aberrant gene expression in sub‐telomeric regions
Source: Aging Cell. 2021 May 21;20(6):e13357. doi: 10.1111/acel.13357 (PMC8208793; doi:10.1111/acel.13357)
Supplement: Supplementary file 1 — Supplementary Material [file ACEL-20-e13357-s001.docx]

**Supplementary Figure Legends**

**Fig. S1 Age-related DEGs as a function of their distance to centromeres.**

Odds ratio was calculated as (No. upregulated DEG in the bin × No. downregulated DEG elsewhere) / (No. upregulated DEG elsewhere × No. downregulated DEG in the bin). Error bars represent 95% confidence intervals.

**Fig. S2 Overlap of age-related DEGs between global and tissue-specific analysis.**

Orange indicates overlapped genes in both global and tissue-specific analysis.Black indicates overlapped genes with opposite effect of regulation, e.g., upregulated in global analysis and downregulated in tissue-specific analysis. Green indicatesgenes which cannot be analyzed in the global analysis because of an average read count <1 across all tissue types, but when analyzing a specific-tissue type has a read count > 1 and was found to be age-related DEG.

**Fig. S3 Odds ratio of age-related upregulated and downregulated DEGs in for each tissue types.**

Only tissues with at least one DEGs were shown. Figures are sorted according to the total numbers of DEGs identified in each tissue. Odds ratio was calculated as (No. upregulated DEG in the bin × No. downregulated DEG elsewhere) / (No. upregulated DEG elsewhere × No. downregulated DEG in the bin). Error bars present 95% confidence intervals.

**Fig. S4 Hi-C loops fromtelomeres mostly end within2Mb from chromosome ends.**

All chromosomal loops in different chromosomes and different cell lines were combined. The circle indicates positions in the genome in the aspect of their distance to chromosome ends. The black lines indicate chromosomal loops.
