# Supplementary material for: Age‐related telomere attrition causes aberrant gene expression in sub‐telomeric regions
Source: Aging Cell. 2021 May 21;20(6):e13357. doi: 10.1111/acel.13357 (PMC8208793; doi:10.1111/acel.13357)
Supplement: Supplementary file 2 — Figure S1. Age‐related DEGs as a function of their distance to centromeres. Figure S2. Overlap of age‐related DEGs between global and tissue‐specific analysis. Figure S3. Odds ratio of age‐related upregulated and downregulated DEGs in for each tissue types. Figure S4. Hi‐C loops fromtelomeres mostly end within 2Mb from chromosome ends. [file ACEL-20-e13357-s003.pptx]

## Slide 1
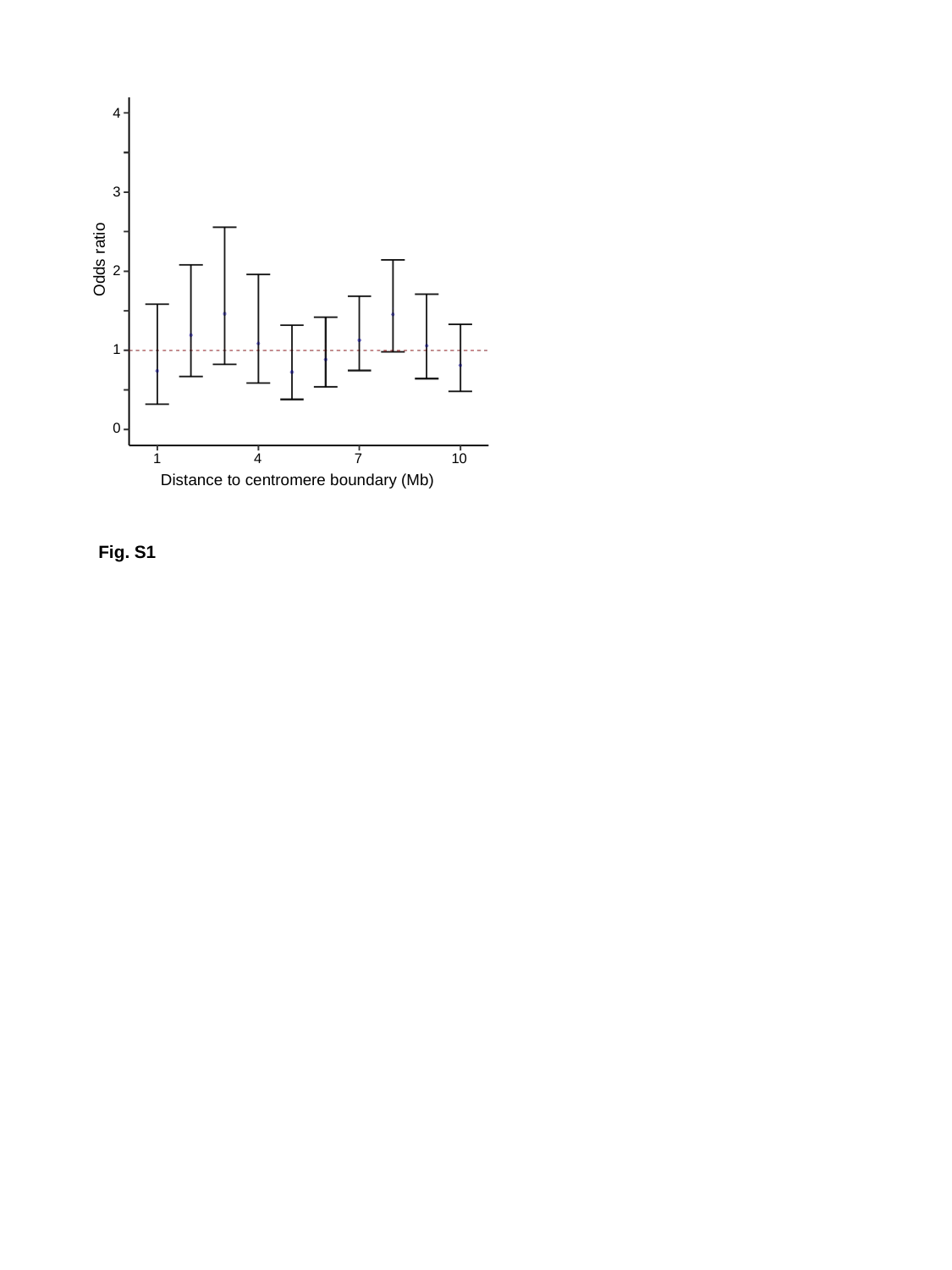

4
3
Odds ratio
2
1
0
1
4
7
10
Distance to centromere boundary (Mb)
Fig. S1

## Slide 2
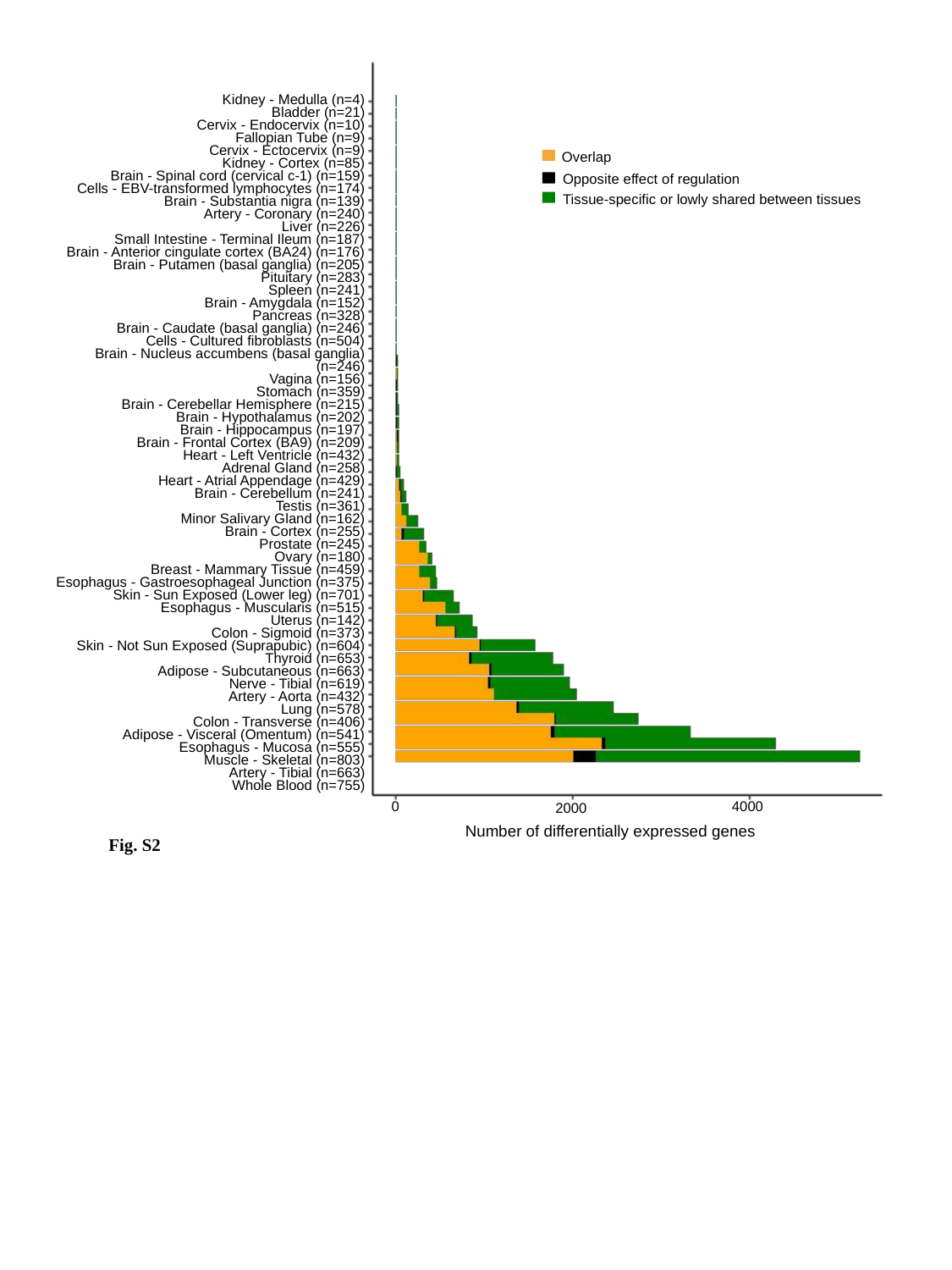

0
4000
2000
Number of differentially expressed genes
Kidney - Medulla (n=4)
Bladder (n=21)
Cervix - Endocervix (n=10)
Fallopian Tube (n=9)
Cervix - Ectocervix (n=9)
Kidney - Cortex (n=85)
Brain - Spinal cord (cervical c-1) (n=159)
Cells - EBV-transformed lymphocytes (n=174)
Brain - Substantia nigra (n=139)
Artery - Coronary (n=240)
Liver (n=226)
Small Intestine - Terminal Ileum (n=187)
Brain - Anterior cingulate cortex (BA24) (n=176)
Brain - Putamen (basal ganglia) (n=205)
Pituitary (n=283)
Spleen (n=241)
Brain - Amygdala (n=152)
Pancreas (n=328)
Brain - Caudate (basal ganglia) (n=246)
Cells - Cultured fibroblasts (n=504)
Brain - Nucleus accumbens (basal ganglia) (n=246)
Vagina (n=156)
Stomach (n=359)
Brain - Cerebellar Hemisphere (n=215)
Brain - Hypothalamus (n=202)
Brain - Hippocampus (n=197)
Brain - Frontal Cortex (BA9) (n=209)
Heart - Left Ventricle (n=432)
Adrenal Gland (n=258)
Heart - Atrial Appendage (n=429)
Brain - Cerebellum (n=241)
Testis (n=361)
Minor Salivary Gland (n=162)
Brain - Cortex (n=255)
Prostate (n=245)
Ovary (n=180)
Breast - Mammary Tissue (n=459)
Esophagus - Gastroesophageal Junction (n=375)
Skin - Sun Exposed (Lower leg) (n=701)
Esophagus - Muscularis (n=515)
Uterus (n=142)
Colon - Sigmoid (n=373)
Skin - Not Sun Exposed (Suprapubic) (n=604)
Thyroid (n=653)
Adipose - Subcutaneous (n=663)
Nerve - Tibial (n=619)
Artery - Aorta (n=432)
Lung (n=578)
Colon - Transverse (n=406)
Adipose - Visceral (Omentum) (n=541)
Esophagus - Mucosa (n=555)
Muscle - Skeletal (n=803)
Artery - Tibial (n=663)
Whole Blood (n=755)
Overlap
Opposite effect of regulation
Tissue-specific or lowly shared between tissues
Fig. S2

## Slide 3
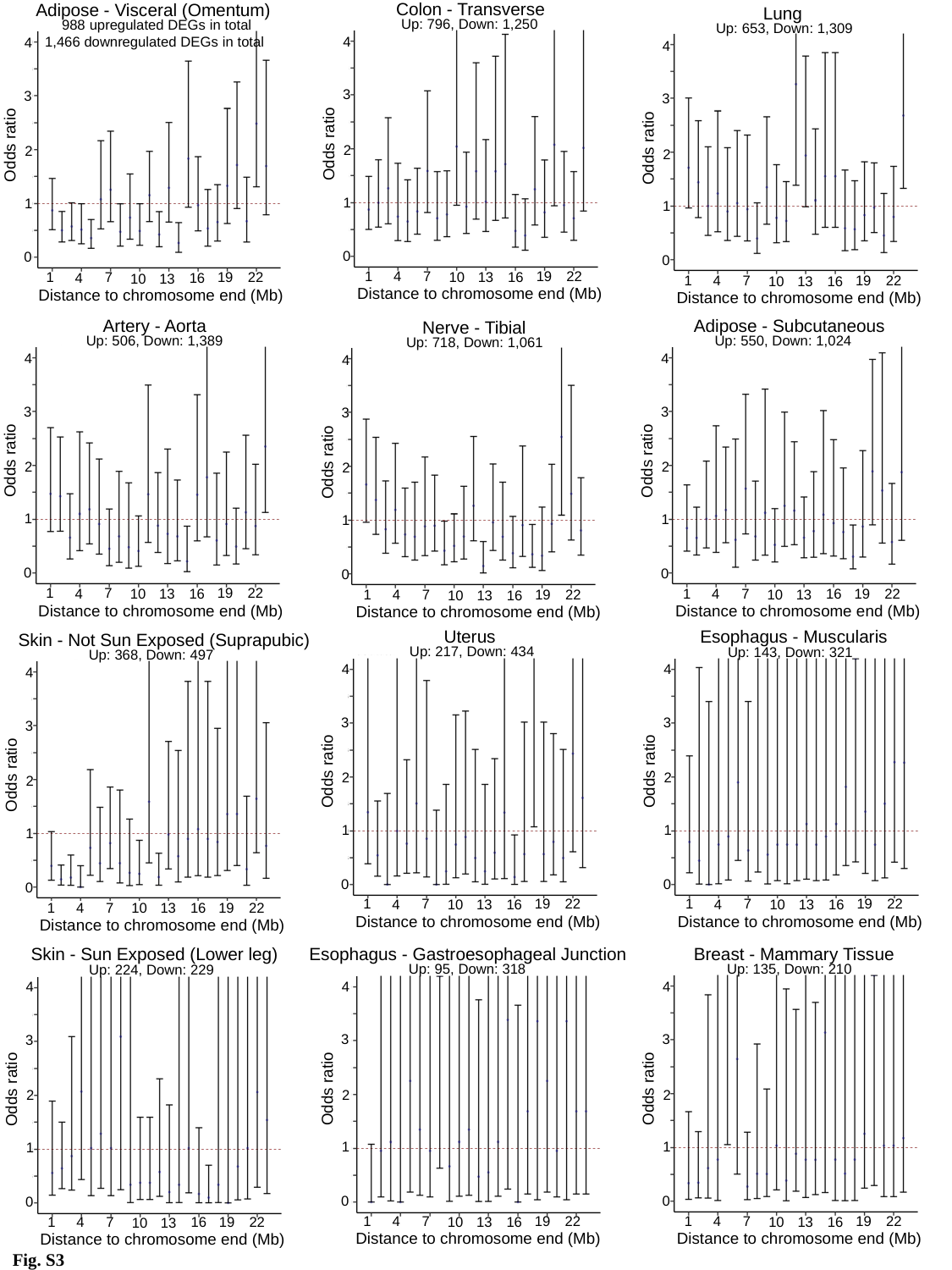

Colon - Transverse
Up: 796, Down: 1,250
4
3
Odds ratio
2
1
0
1
7
19
22
4
16
10
13
Distance to chromosome end (Mb)
Adipose - Visceral (Omentum)
988 upregulated DEGs in total
1,466 downregulated DEGs in total
4
3
Odds ratio
2
1
0
1
7
19
22
4
16
10
13
Distance to chromosome end (Mb)
Lung
Up: 653, Down: 1,309
4
3
Odds ratio
2
1
0
1
7
19
22
4
16
10
13
Distance to chromosome end (Mb)
Artery - Aorta
Up: 506, Down: 1,389
4
3
Odds ratio
2
1
0
1
7
19
22
4
16
10
13
Distance to chromosome end (Mb)
Adipose - Subcutaneous
Up: 550, Down: 1,024
4
3
Odds ratio
2
1
0
1
7
19
22
4
16
10
13
Distance to chromosome end (Mb)
Nerve - Tibial
Up: 718, Down: 1,061
4
3
Odds ratio
2
1
0
1
7
19
22
4
16
10
13
Distance to chromosome end (Mb)
Uterus
Up: 217, Down: 434
4
3
Odds ratio
2
1
0
1
7
19
22
4
16
10
13
Distance to chromosome end (Mb)
Esophagus - Muscularis
Up: 143, Down: 321
4
3
Odds ratio
2
1
0
1
7
19
22
4
16
10
13
Distance to chromosome end (Mb)
Skin - Not Sun Exposed (Suprapubic)
Up: 368, Down: 497
4
3
Odds ratio
2
1
0
1
7
19
22
4
16
10
13
Distance to chromosome end (Mb)
Esophagus - Gastroesophageal Junction
Up: 95, Down: 318
4
3
Odds ratio
2
1
0
1
7
19
22
4
16
10
13
Distance to chromosome end (Mb)
Breast - Mammary Tissue
Up: 135, Down: 210
4
3
Odds ratio
2
1
0
1
7
19
22
4
16
10
13
Distance to chromosome end (Mb)
Skin - Sun Exposed (Lower leg)
Up: 224, Down: 229
4
3
Odds ratio
2
1
0
1
7
19
22
4
16
10
13
Distance to chromosome end (Mb)
Fig. S3

## Slide 4
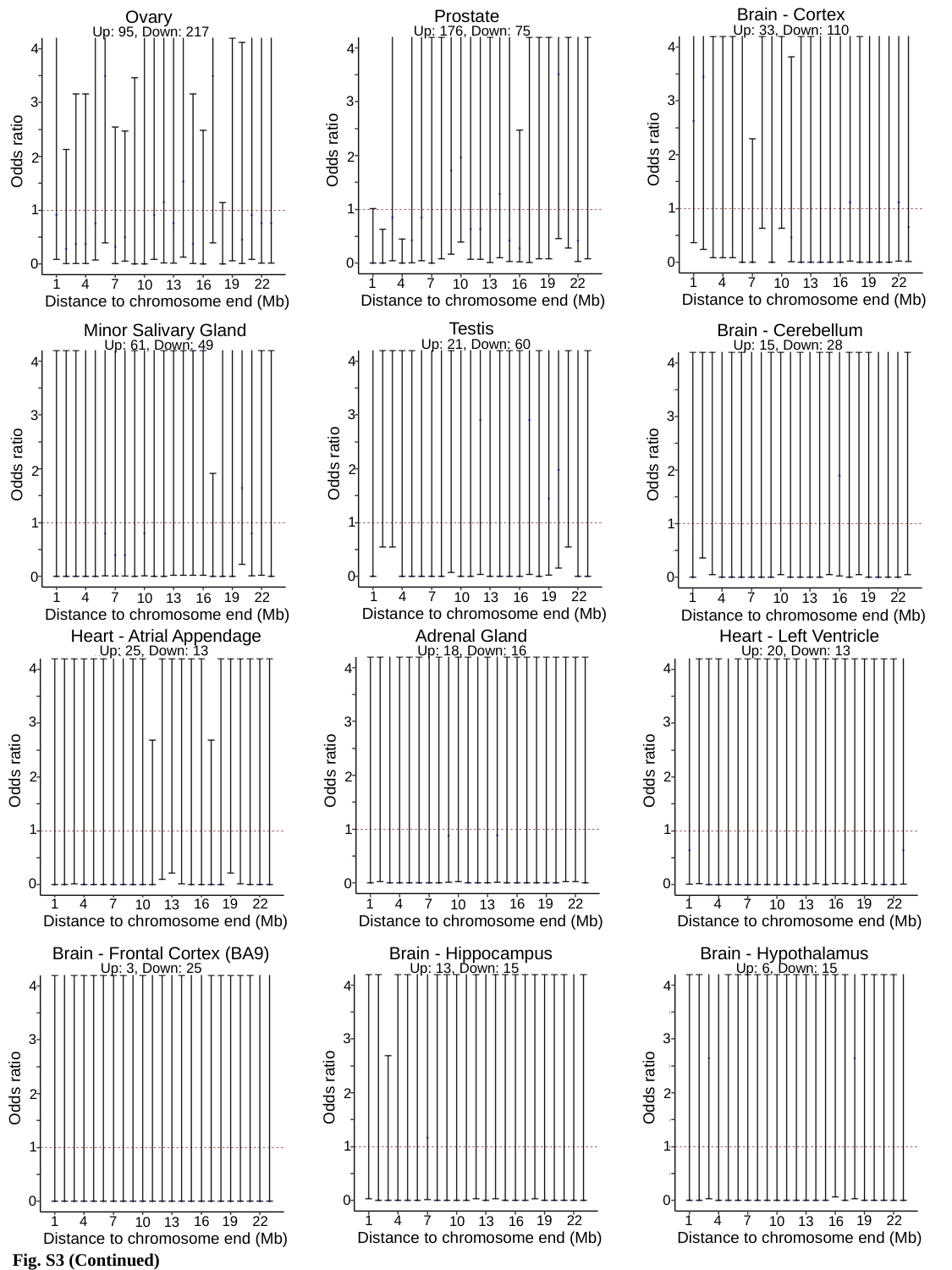

Brain - Cortex
Up: 33, Down: 110
4
3
Odds ratio
2
1
0
1
7
19
22
4
16
10
13
Distance to chromosome end (Mb)
Prostate
Up: 176, Down: 75
4
3
Odds ratio
2
1
0
1
7
19
22
4
16
10
13
Distance to chromosome end (Mb)
Ovary
Up: 95, Down: 217
4
3
Odds ratio
2
1
0
1
7
19
22
4
16
10
13
Distance to chromosome end (Mb)
Testis
Up: 21, Down: 60
4
3
Odds ratio
2
1
0
1
7
19
22
4
16
10
13
Distance to chromosome end (Mb)
Minor Salivary Gland
Up: 61, Down: 49
4
3
Odds ratio
2
1
0
1
7
19
22
4
16
10
13
Distance to chromosome end (Mb)
Brain - Cerebellum
Up: 15, Down: 28
4
3
Odds ratio
2
1
0
1
7
19
22
4
16
10
13
Distance to chromosome end (Mb)
Adrenal Gland
Up: 18, Down: 16
4
3
Odds ratio
2
1
0
1
7
19
22
4
16
10
13
Distance to chromosome end (Mb)
Heart - Atrial Appendage
Up: 25, Down: 13
4
3
Odds ratio
2
1
0
1
7
19
22
4
16
10
13
Distance to chromosome end (Mb)
Heart - Left Ventricle
Up: 20, Down: 13
4
3
Odds ratio
2
1
0
1
7
19
22
4
16
10
13
Distance to chromosome end (Mb)
Brain - Hippocampus
Up: 13, Down: 15
4
3
Odds ratio
2
1
0
1
7
19
22
4
16
10
13
Distance to chromosome end (Mb)
Brain - Hypothalamus
Up: 6, Down: 15
4
3
Odds ratio
2
1
0
1
7
19
22
4
16
10
13
Distance to chromosome end (Mb)
Brain - Frontal Cortex (BA9)
Up: 3, Down: 25
4
3
Odds ratio
2
1
0
1
7
19
22
4
16
10
13
Distance to chromosome end (Mb)
Fig. S3 (Continued)

## Slide 5
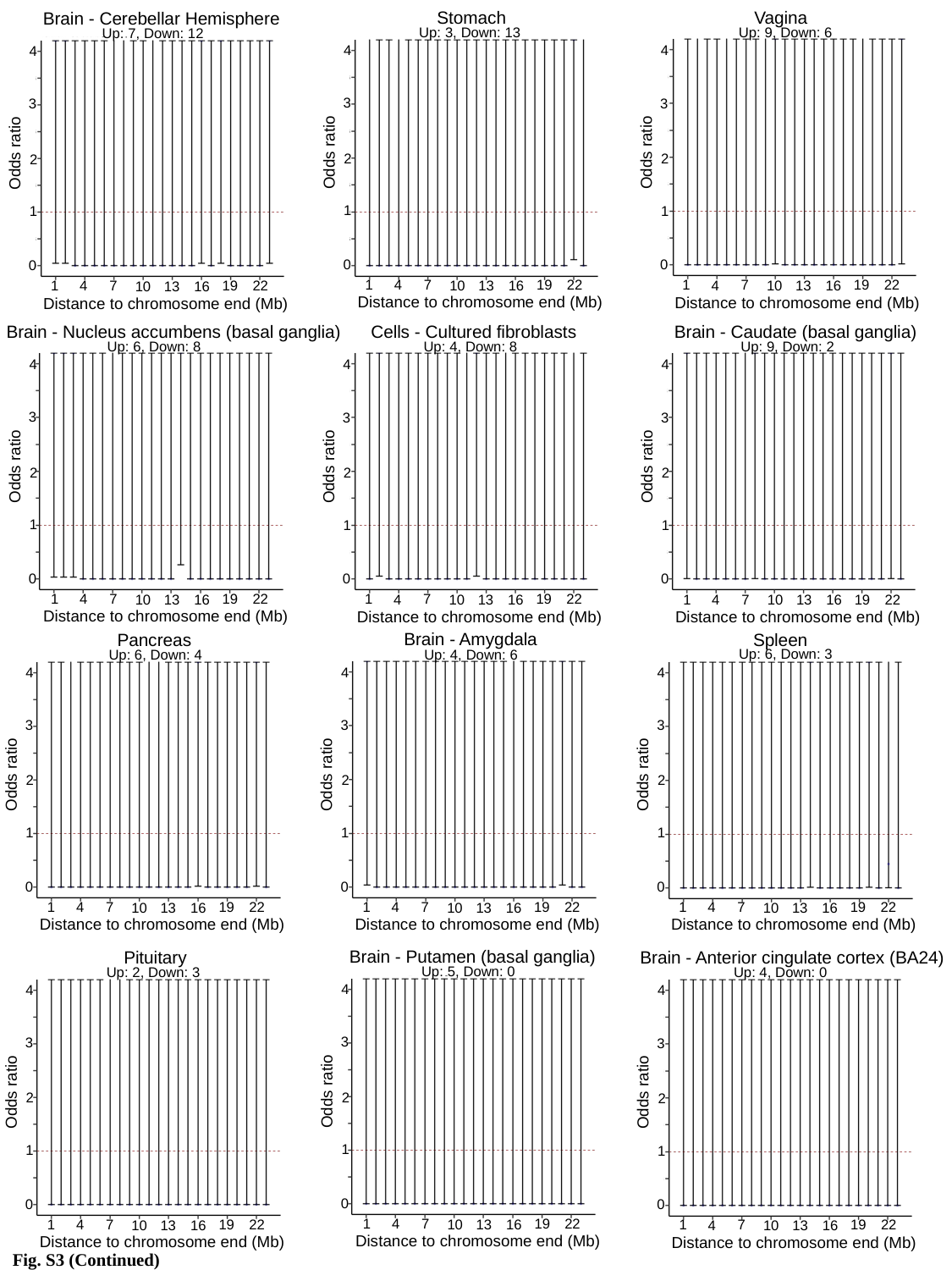

Stomach
Up: 3, Down: 13
4
3
Odds ratio
2
1
0
1
7
19
22
4
16
10
13
Distance to chromosome end (Mb)
Vagina
Up: 9, Down: 6
4
3
Odds ratio
2
1
0
1
7
19
22
4
16
10
13
Distance to chromosome end (Mb)
Brain - Cerebellar Hemisphere
Up: 7, Down: 12
4
3
Odds ratio
2
1
0
1
7
19
22
4
16
10
13
Distance to chromosome end (Mb)
Brain - Nucleus accumbens (basal ganglia)
Up: 6, Down: 8
4
3
Odds ratio
2
1
0
1
7
19
22
4
16
10
13
Distance to chromosome end (Mb)
Cells - Cultured fibroblasts
Up: 4, Down: 8
4
3
Odds ratio
2
1
0
1
7
19
22
4
16
10
13
Distance to chromosome end (Mb)
Brain - Caudate (basal ganglia)
Up: 9, Down: 2
4
3
Odds ratio
2
1
0
1
7
19
22
4
16
10
13
Distance to chromosome end (Mb)
Brain - Amygdala
Up: 4, Down: 6
4
3
Odds ratio
2
1
0
1
7
19
22
4
16
10
13
Distance to chromosome end (Mb)
Pancreas
Up: 6, Down: 4
4
3
Odds ratio
2
1
0
1
7
19
22
4
16
10
13
Distance to chromosome end (Mb)
Spleen
Up: 6, Down: 3
4
3
Odds ratio
2
1
0
1
7
19
22
4
16
10
13
Distance to chromosome end (Mb)
Brain - Putamen (basal ganglia)
Up: 5, Down: 0
4
3
Odds ratio
2
1
0
1
7
19
22
4
16
10
13
Distance to chromosome end (Mb)
Pituitary
Up: 2, Down: 3
4
3
Odds ratio
2
1
0
1
7
19
22
4
16
10
13
Distance to chromosome end (Mb)
Brain - Anterior cingulate cortex (BA24)
Up: 4, Down: 0
4
3
Odds ratio
2
1
0
1
7
19
22
4
16
10
13
Distance to chromosome end (Mb)
Fig. S3 (Continued)

## Slide 6
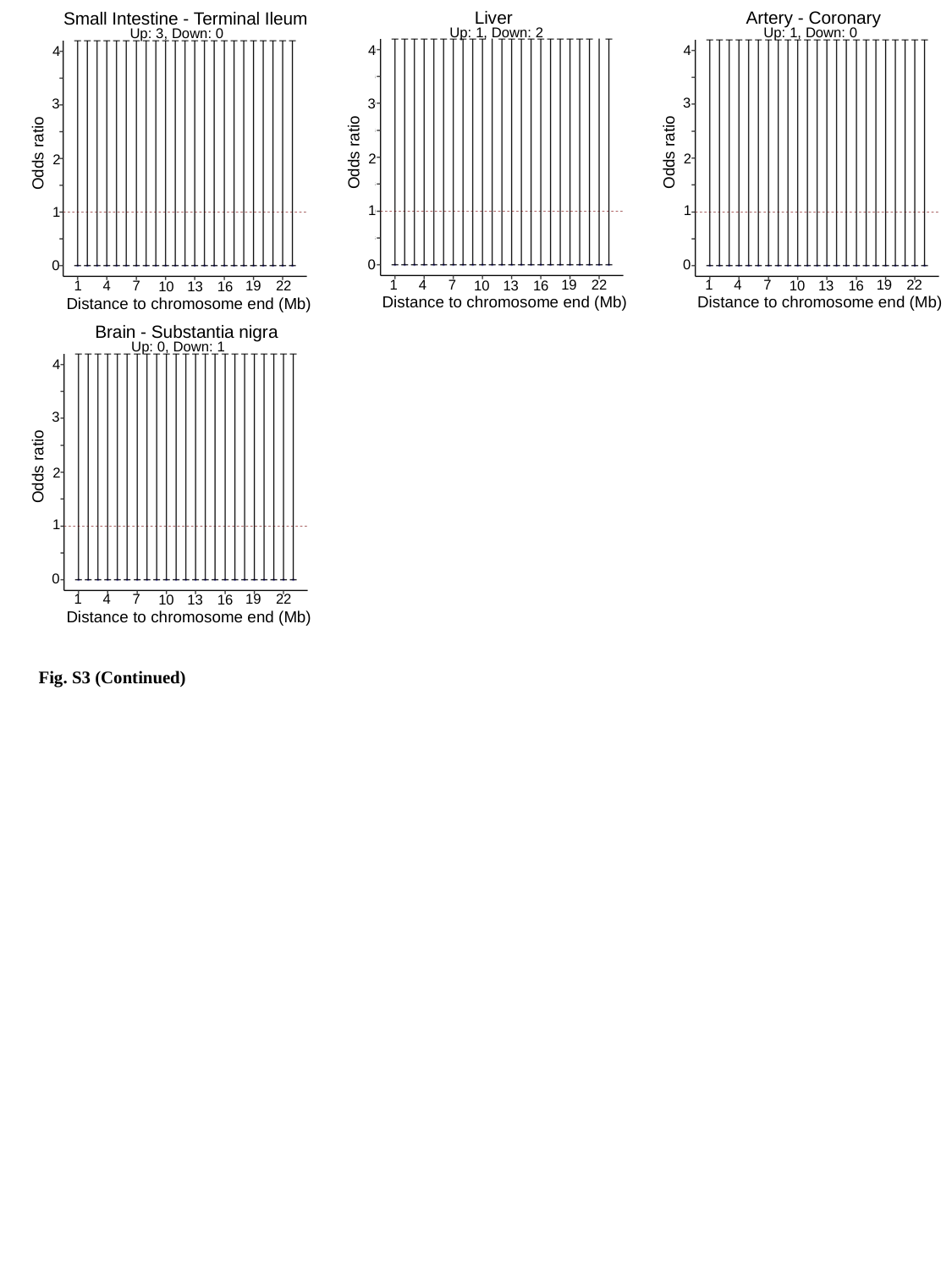

Artery - Coronary
Up: 1, Down: 0
4
3
Odds ratio
2
1
0
1
7
19
22
4
16
10
13
Distance to chromosome end (Mb)
Liver
Up: 1, Down: 2
4
3
Odds ratio
2
1
0
1
7
19
22
4
16
10
13
Distance to chromosome end (Mb)
Small Intestine - Terminal Ileum
Up: 3, Down: 0
4
3
Odds ratio
2
1
0
1
7
19
22
4
16
10
13
Distance to chromosome end (Mb)
Brain - Substantia nigra
Up: 0, Down: 1
4
3
Odds ratio
2
1
0
1
7
19
22
4
16
10
13
Distance to chromosome end (Mb)
Fig. S3 (Continued)

## Slide 7
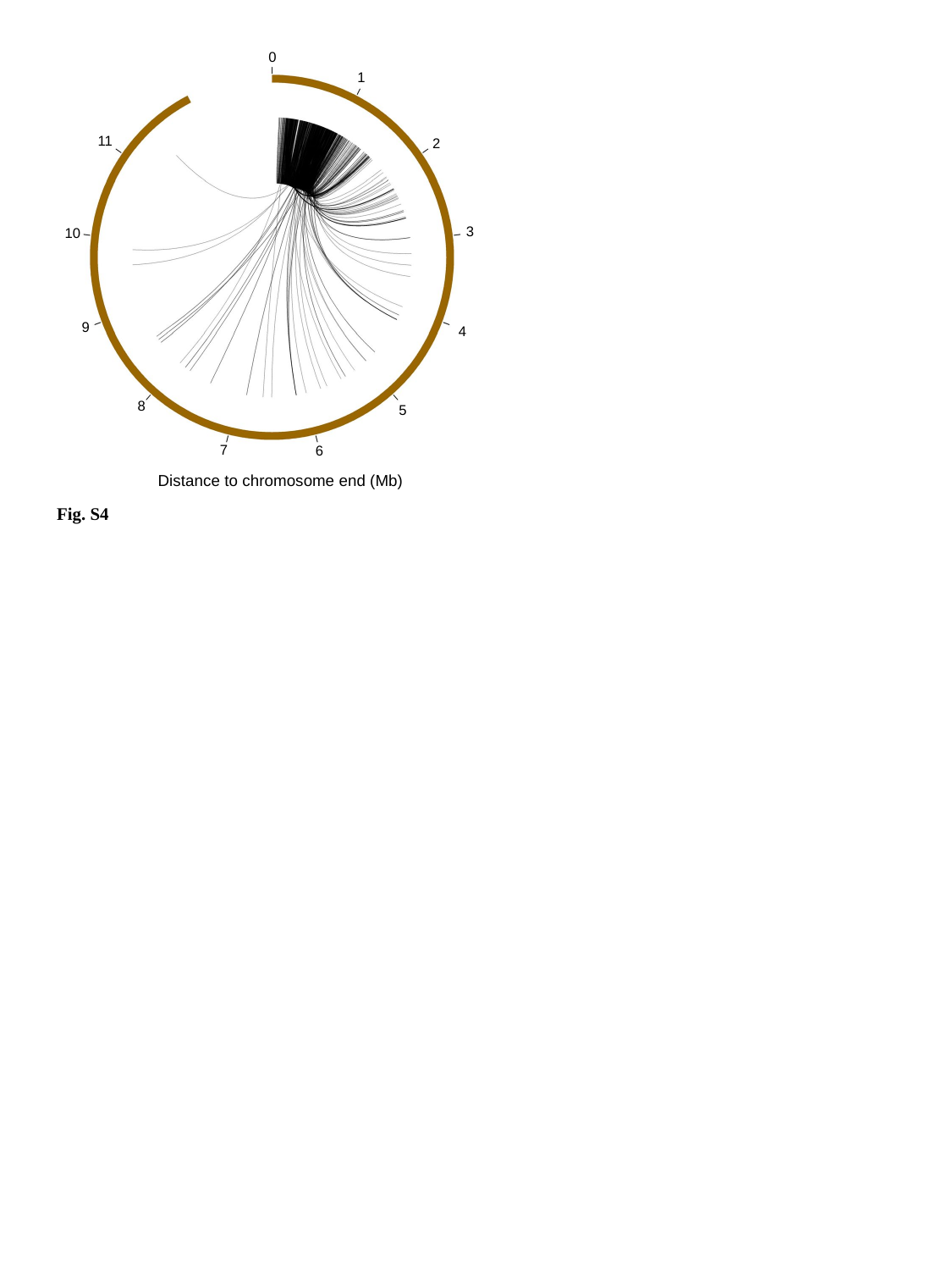

0
1
11
2
3
10
9
4
8
5
7
6
Distance to chromosome end (Mb)
Fig. S4
